# Advancements in xenotransplantation: paving the way for revolutionary developments in reconstructive plastic surgery

**DOI:** 10.1590/acb393424

**Published:** 2024-07-15

**Authors:** Vitor Penteado Figueiredo Pagotto, Fábio de Freitas Busnardo, Silvano Mário Attílio Raia, Rolf Gemperli

**Affiliations:** 1 Universidade de São Paulo – Faculdade de Medicina – Hospital das Clínicas – São Paulo (SP), Brazil.; 2 Instituto do Câncer do Estado de São Paulo – São Paulo (SP), Brazil.; 3 Academia Nacional de Medicina – Rio de Janeiro (RJ), Brazil.

The global landscape of organ transplantation is undergoing a paradigm shift, with an escalating focus on xenotransplantation–a groundbreaking frontier in medical science. This editorial delves into the burgeoning importance of xenotransplantation studies, particularly within the realm of reconstructive plastic surgery, and highlights a significant opportunity for further exploration.

Organ allotransplantation, while transformative, grapples with an inherent ceiling–limited human donor organs^1^^–^ 3. The current demand for organ transplants far exceeds the available supply, leaving over 100,000 individuals on national transplant waiting lists in the United States alone. Xenotransplantation emerges as a promising solution to this critical shortage, with recent human decedents experiments showcasing initial success in pig-to-human heart and kidney xenotransplantation. The potential to overcome morbidity, mortality, and cost challenges makes xenotransplantation a beacon of hope.

A recent case involved a 57-year-old man with nonischemic cardiomyopathy, dependent on extracorporeal membrane oxygenation (ECMO)^4^^,^ 5. Despite being ineligible for traditional allografts, he received a heart from a genetically modified pig with ten individual gene edits. The patient, initially weaned from ECMO, experienced sudden complications on day 49 post-transplantation, prompting further studies to unravel the mechanisms behind these changes. This case underscores the need for continued research to enhance our understanding of xenotransplantation outcomes.

Skin xenotransplantation, a focus within reconstructive plastic surgery, has undergone significant evolution. Early xenografts faced rapid rejection, but recent advances in swine-to-non-human primate models show xenografts surviving up to 30 days without maintenance immunosuppression^6^^,^ 7. These developments open new possibilities for treating patients with extensive burns, offering living, functional skin xenotransplants with clinically relevant shelf-lives. The question of whether genetically modified pig skin will be limited to temporary dressings or utilized in more intricate flaps, such as those in face transplants necessitating immunosuppression, remains a challenging inquiry that warrants further investigation.

The narrative of xenotransplantation extends beyond organs to human skin, and studies have harnessed the power of xenografting for in-vivo analysis. Humanized animals, through human-to-mouse xenografting, facilitate real-time evaluation of physiological processes, clonal analysis, genetic modifications, and drug discovery–providing insights that animal models and culture systems alone cannot replicate^8^.

In line with this transformative landscape, the São Paulo Research Foundation (Fundação de Amparo à Pesquisa do Estado de São Paulo – FAPESP) announces a prestigious postdoctoral fellowship at the Universidade de São Paulo’s Center for Xenotransplantation Studies (Table 1). This opportunity invites researchers to contribute to the cutting-edge field of xenotransplantation, with a particular focus on skin grafts and their implications for reconstructive plastic surgery. The fellowship seeks to attract scholars committed to advancing medical knowledge and addressing the challenges outlined in recent xenotransplantation studies.

**Table 1:** Postdoctoral fellowship orientations.

Project
Evaluation of Hyperacute Rejection in Genetically Modified Pig Skin Transplantation in Human Models and Its Implications in Xenotransplantation
Candidates should have: • Experience in tissue engineering and microsurgery • Dissection and assessment of hyperacute rejection • Development of cryopreservation model
Application • Curriculum Vitae (according to FAPESP guidelines, at fapesp.br/sumula) • Letter of Motivation • PhD Completion Certificate
Send the following documents in PDF format the emails: silvanoraia@uol.com.br and ccdxenotransplante@gmail.com with subject: Selection-PD-Skin

Source: elaborated by the authors.

As the momentum in xenotransplantation research builds, it becomes imperative for the global medical community to unite in the pursuit of knowledge and innovation. The developments in skin xenotransplantation and the establishment of a postdoctoral fellowship underscore the immense potential that xenotransplantation holds for the future of medicine, particularly within the domain of reconstructive plastic surgery. As we embark on this transformative journey, collaboration and commitment will be the cornerstones of ushering in a new era in medical science.

## Data Availability

Data sharing is not applicable.

## References

[1] Carrier AN, Verma A, Mohiuddin M, Muller YD, Bhati C, Buhler LH, Maluf DG, Meier RPH (2022). Xenotransplantation: A New Era. Front Immunol.

[2] Fischer K, Schnieke A (2022). Xenotransplantation becoming reality. Transgenic Res.

[3] Raia SMA (2022). Xenotransplantation: a consistent perspective. Rev Col Bras Cir.

[4] Griffith BP, Goerlich CE, Singh AK, Rothblatt M, Lau CL, Shah A, Lorber M, Grazioli A, Saharia KK, Hong SN, Joseph SM, Ayares D, Mohiuddin MM (2022). Genetically Modified Porcine-to-Human Cardiac Xenotransplantation. N Engl J Med.

[5] Cooper DKC, Kobayashi T (2024). Xenotransplantation experiments in brain-dead human subjects–A critical appraisal. Am J Transplant.

[6] Kalsi R, Messner F, Brandacher G (2020). Skin xenotransplantation: technological advances and future directions. Curr Opin Organ Transplant.

[7] Cherkashina OL, Morgun EI, Rippa AL, Kosykh A, Alekhnovich AV, Stoliarzh AB, Terskikh VV, Vorotelyak EA, Kalabusheva EP (2023). Blank Spots in the Map of Human Skin: The Challenge for Xenotransplantation. Int J Mol Sci.

[8] Montgomery RA, Griesemer AD, Segev DL, Sommer P (2024). The decedent model: A new paradigm for de-risking high stakes clinical trials like xenotransplantation. Am J Transplant.

